# Simulation of the biocide distribution in soil using PELMO coupled with COMLEAM

**DOI:** 10.1007/s11356-024-35760-y

**Published:** 2025-01-09

**Authors:** Nadine Kiefer, Judith Klein, Mirko Rohr, Matthias Noll, Michael Burkhart, Michael Klein, Stefan Kalkhof

**Affiliations:** 1 Institute for Bioanalysis, University of Applied Sciences Coburg, Coburg, Germany; 2 Fraunhofer Institute for Molecular Biology and Applied Ecology IME, Schmallenberg, Germany; 3 Institute of Environmental and Process Engineering (UMTEC), OST - Eastern Switzerland University of Applied Scienes, Rapperswil, Switzerland; 4 Proteomics Unit, Fraunhofer Institute for Cell Therapy and Immunology, Leipzig, Germany; 5 Institute of Analytical Chemistry, Faculty of Chemistry and Mineralogy, University of Leipzig, Leipzig, Germany

**Keywords:** Simulation, COMLEAM, PELMO, Biocides, Soil, Building materials, Risk assessment

## Abstract

Biocides, applied in building materials as antimicrobial protectants, can be leached out by rain, presenting substantial environmental risks as confirmed by studies on aquatic environments. However, these biocides are consistently released throughout the year in a diluted form, posing unique challenges for the prediction of transport, transformation, and ecotoxicity assessment in soil. To address this challenge, we combined COMLEAM, which predicts leaching from facades into the soil, with the FOCUS PELMO pesticide model to predict biocide distribution in soil. The study predicted the concentration of the commonly used biocides 1,2-benzisothiazol-3(2H)-one (BIT), 2-n-octyl-4-isothiazolinone-3-one, and terbutryn at various soil depths over 600 days, thereby evaluating the influence of different leaching simulations, absorption, and degradation kinetics on the PELMO simulation results. The findings suggest an effective simulation of biocide behavior in soil, with a tendency to overestimate the risk associated with short-acting in-can preservatives like BIT and provide more precise predictions for long-acting film preservatives. Biocide concentrations showed little variance in the upper layers and increased discrepancies at greater depths. This integrated approach provides a cost-effective means for predicting environmental risks and formulating management strategies, especially when paired with experimentally determined soil-specific degradation and absorption values.

**Supplementary Information:**

The online version contains supplementary material available at 10.1007/s11356-024-35760-y.

## Introduction

Biocides are commonly used in building materials to extend product life and protect buildings from microbial growth and biological destruction (Reiß et al. 2021). To achieve this goal against a wide range of different microorganisms, a render or paint product typically contains a mixture of three to five biocides, resulting in an estimated total biocide content of 0.5% w/w (Burkhardt et al. 2011). Biocide formulations are used for two different purposes. In-can preservatives are added to prevent microbial growth during storage and film preservatives can be used to provide long-term protection after the facade has been built (Reiß et al. 2021). Typically, low molecular weight and more hydrophilic isothiazolinones are used as in-can preservatives, while hydrophobic substances such as higher molecular weight isothiazolinones, phenylureas, triazines, and carbamates are used as film preservatives (Kiefer et al. 2023). However, these biocides can leach out during rain events and end up in the environment. Indeed, biocides from building materials have been detected in aquatic environments (Burkhardt et al. 2012; Wittmer et al. 2011). Thus, there is clear evidence that biocide-containing building materials are an additional source of environmental pollution (Paijens et al. 2020).

Depending on the building design, biocides can also leach into the surrounding soil (Bollmann et al. 2014). Exposure to the aquatic environment is well studied (Paijens et al. 2020), but only a few experimental studies exist examining the terrestrial environment (Bollmann et al. 2017; Linke et al. 2023). Some of these biocides applied in building materials have also been used in agriculture and have therefore been extensively studied for soil contamination. These findings are transferable to some extent (Wittmer et al. 2011), however significant differences in application and leaching scenarios must be taken into account. To address these differences a COnstruction Material LEAching Model (COMLEAM) was developed (2022a) to estimate the biocide leaching from buildings (Linke et al. 2022; Linke et al. 2023).

Agricultural biocides are only applied in defined, high doses on a few dates per year. In contrast, biocides leached from the various building materials are released uncontrollably in large dilutions during rain events throughout the whole year (Bollmann et al. 2017; Reiß et al. 2021). The leaching process and the distribution, accumulation, and degradation in the soil are multi-parametric and complex scenarios. To address all these variable parameters and obtain an adaptive scenario, some researchers try to simulate the expected concentration of biocides in leachate, soil, and water to estimate the risk potential of biocides to the environment (Bandow et al. 2020; Moser et al. 2018; Schoknecht et al. 2022; Vega-Garcia et al. 2022; Wittmer et al. 2016). Furthermore, the dilution of the applied concentrations differs. In the pesticide scenario, there is a larger time gap between substance application and rain dilution. In the biocide scenario, the substance leaching is directly related to rain events. Due to the higher dilution, this may result in higher concentrations in deeper soil layers because of the short adsorption time and faster transport through the soil layers. Accordingly, these differences must be considered in assessments and predictions of their bioavailability and terrestrial ecotoxicity (Reiß et al. 2021).

To estimate the transport of substances through unsaturated soil, 1D FOCUS models like PELMO are already used for the regulation of pesticides (FOCUS 2000, 2006, 2009/2014; Klein 1995). Critical parameters for the PELMO simulation need to be checked for sensibility upon adaption of the model for the biocide scenario. Herein the distribution of a substance in soil, degradation, and adsorption are critical parameters. (Dubus et al. 2003; Klein et al. 2000; Pantelelis et al. 2006; Schoknecht et al. 2022; Vega-Garcia et al. 2022). The parameters that influence how pesticides are sorbed and degraded in soils are extensively reviewed by Arias-Estévez et al. (2008). They are determined by a large number of factors that can be classified as climatic parameters, the physico-chemical properties of the substances, the properties of the soil, and the experimental setting in general. Temperature, precipitation quantity, relative humidity, and evaporation constitute the main climate parameters (Reiß et al. 2021). Important soil characteristics include vegetation cover, geography, microbial community composition and abundance, contaminants, and soil structure. The texture of the soil includes the clay content, structure, organic matter, pH, soil moisture, and the content of mineral ions. The substance properties such as concentration, solubility, and chemical nature, and application mode such as time, amount, and frequency form critical parameters for the biocide distribution in soil (Reiß et al. 2021).

As an example, the influence of the soil properties on the behavior of a biocide, isoproturon, in soil was studied in detail. For isoproturon, it was shown, that the half-life varies from 4 to 200 days depending on the soil properties, such as organic matter content, pH, nutrient status (N, P, K), and soil microbial biomass (Walker et al. 2001). Other research groups found half-lives for isoproturon to be ranging from 31 to 483 days in 25 soil samples obtained from different areas (Beck et al. 1996), which points to the high impact of experimental conditions, soil types, and the presence of degrading microbial community members (Bending et al. 2003). It should be noted that urban soils can be very different from agricultural soils in terms of structure, pH, and biodiversity, and therefore may have an even lower potential for degradation. In line with a study performed by Bollmann et al. (2017) on biocides of different chemical groups used in building materials, it was defined that biocides differ significantly in their degradation and persistence behavior in (sub-)urban soil.

The degradation and adsorption of phenoxy alkanoic acid herbicides were reviewed by Paszko et al. (2016), where microbial degradation was identified to be the most important driving parameter for degradation rates in soil. With regard to the adsorption behavior, two critical parameters were identified as acid dissociation constants (pKa) and n-octanol–water partition coefficients (log *P*). The highest adsorption tendency corresponded to the highest pKa and log *P* values. These form a general directive, while the Kd value varies depending on the soil properties (Paszko et al. 2016).

Finally, some biocides and degradation products thereof can accumulate in soil (Bollmann et al. 2017). Due to the fact, that the environmental penetration of biocide occurs continuously over the year with every rain event, this accumulation is not controllable, as opposed to the agricultural use. Therefore, the toxicity assessments of biocide accumulation over the long term need to be studied and simulated, as well as the impact of variable parameters such as application scenario, degradation kinetics, and adsorption/desorption values on soil depth depended on concentrations.

Keeping with the above, we combined the pesticide regulatory model FOCUS PELMO (prediction of distribution and degradation of biocides in soil) with the biocide release simulation software COMLEAM to identify a scenario for the risk assessment of leaching biocides from facades into the soil. Using this setup, we predicted the resulting soil depth-dependent concentrations over a period of 600 days on the model of frequently applied biocides 1,2-benzisothiazol-3(2H)-one (BIT), 2-n-octyl-4-isothiazolinone-3-one (OIT), and terbutryn (TB). We evaluated the extent to which the different application scenarios (predicted vs. measured biocide release in a natural-weathering experiment compared to a DIN Immersion Scenario) and the adsorption and degradation kinetics (experimentally determined vs. obtained from literature) affect the resulting courses of the PELMO simulations, as well as the obtained maximal biocide concentrations in the soil layer.

## Materials and methods

### Materials

Methanol (MeOH) (HPLC gradient quality (X948.2) and LC–MS quality (AE71.1)) and formic acid (LC–MS quality (1EHK.1)) were used as solvents (Carl Roth GmbH&Co.KG, Germany). Analytical standards for OIT (2-n-octyl-4-isothiazolinone-3-one; lot: G1112533), BIT (1,2-benzisothiazol-3(2H)-one; G1165715), and TB (tebutryn; lot: G1008790) were purchased from Dr. Ehrenstorfer GmbH (Augsburg, Germany). All solutions were prepared using Milli-Q water (18.2 MΩ) and/or MeOH (LC–MS quality). The reference sol Refesol 02-A was provided by Fraunhofer IME (Schmallenberg, Germany).

### Study design

**Fig. 1:**
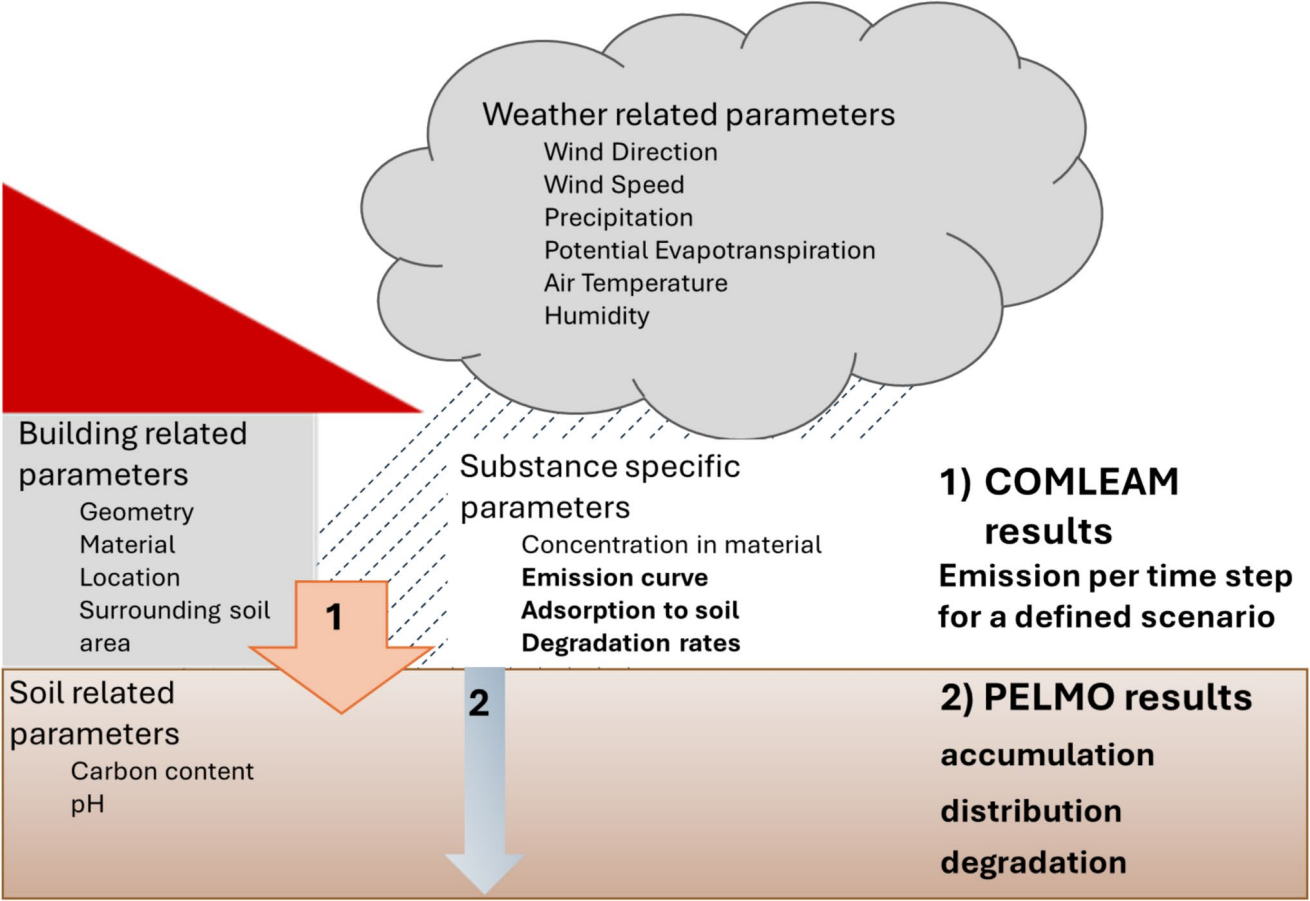
Schematic presentation of the parameters influencing the simulations and the expected output. On the left side, the main parameters which influence the simulation are presented. The bold parameters are discussed in this work. On the right side, the simulation program and the information output are shown

COMLEAM and PELMO were used in combination to estimate the biocide risk originating from building materials in soil. As input parameters substance, weather, and/or soil data were collected either experimentally or from the literature performing these simulations (Fig. 1, Supplement Fig. A1).

### Experimental determination of adsorption, desorption, and degradation of biocides in the reference soil

For the simulations, the distribution, degradation, and persistence in the soil of the three active substances BIT (1,2-benzothiazole-3 (2H)-one), OIT (octhilinone), and TB (TB) were simulated. For this purpose, the physiochemical properties of the biocides, as well as the soil-related parameters, namely adsorption and degradation were used as input parameters.

#### Soil parameters

In order to ensure a well-characterized soil with reproducible parameters for the experiments, RefeSol 02-A was obtained from the Fraunhofer IME (Schmallenberg, Germany). RefeSol 02-A is a biocide-free, silt loam, sub-acidic, light humic stannic luvisol, representative of the Dübendorf area in Switzerland, where the natural weather experiment used in the simulation occurred. The soil characteristics, detailed in Supplement Table A1, were utilized to conduct the simulations.

#### Adsorption values

The adsorption values were determined according to the Organization for Economic Cooperation and Development (OECD) Guideline Number 106 (OECD 2000). The OECD Guideline protocol was adapted to the reference soil (RefeSoil02A) and was performed by Parallel Method, i.e. each sample is placed in a separate sample vial. As described in the guideline, the optimal ratio between the soil and aqueous phase was determined experimentally with regard to the optimal adsorption behavior. The mixing ratios of soil to aqueous phase 1:10, 1:4, and 1:2 (w/v) were tested for their adsorption behavior. The soil was pre-equilibrated with a 0.01 M calcium chloride solution using a horizontal shaker, then the 2.0 mg L^−1^ biocide solutions were added and samples were collected after 4, 8, 24, and 48 h.

The soil/ aqueous phase ratio was determined to be 1:10, samples were taken at different time points (2, 4, 6, 8, and 24 h) at the same biocide concentration. Longer incubation time was used for BIT, with an additional sampling time point at 48 h. Desorption kinetics were determined similar to adsorption kinetics at a constant concentration (2.0 mg L^−1^). Samples were pre-equilibrated for 12 h with 0.1 molar calcium chloride solution. Adsorption and desorption isotherms were determined differently, focusing on the fixed time point (48 h) with different biocide concentrations (1.5, 2.0, and 3.0 mg L^−1^). Upon the corresponding incubation time samples were prepared for analysis as described below.

Afterward, the samples were prepared for the analysis by centrifuging two times (5000 rpm, 10 °C, 30 min), the supernatant was collected, and a solid phase extraction (SPE) was used to desalt the sample. At the beginning, the SPE column (Cormabond C18 Hydra, Carl Roth, Germany) was washed and equilibrated twice with 5 mL MeOH, and twice with 5 mL MilliQ water. Afterwards, 5 mL of the supernatant was added. For the following desalting steps, 5 mL MilliQ water was used twice. For the biocide elution step, 2 mL MeOH was used. Finally, the collected samples were filtered (0.2 µm polyamide filter) and transferred into HPLC vials.

For the Quantification of the biocides, an HPLC equipped with an UV/VIS detector was used; the method was described by Kiefer et al. (2023).

#### Degradation studies

To determine the half-lives of the biocide (DT_50_ values), degradation studies were conducted over a 28-day period using RefeSoil02A. Biocide soil mixtures were prepared as follows: 10 mg kg^−1^ of single biocide, overall 10 mg kg^−1^ of biocide mixture (2.5 mg each), and 40 mg kg^−1^ of biocide mixture (10 mg each) were added into the reference soil. For this purpose, 10% of the complete amount of soil was weighed and covered with 25 mL of 100 mg L^−1^ biocide/MeOH solution. The MeOH was evaporated overnight, and the 25 g of the spiked soil was mixed with 225 g of the remainder of the soil sample. The moisture of the soil sample was adjusted to 50% of its water holding capacity (WHC) by adding deionized water. At each sampling date, five replicates of the soil were weighted (3–5 g) into reaction vials with low binding capacity (SARSTEDT AG, Nümbrecht, Germany) and covered with parafilm. To maintain air circulation, holes were cut in the parafilm. Each centrifugation tube was weighed and the water content of the soil was controlled by weighing weekly.

The biocides were re-extracted from the soil samples using ultrasonic extraction, the method previously described by Reiß et al. (2024). An HPLC equipped with a UV/VIS detector (Waters Corporation Milford, MA, USA) was used to analyze the eluates (Kiefer et al. 2023). Finally, degradation curves were calculated and the DT_50_ values were determined.

### Weather data

Weather data used were recorded in the Dübendorf (Switzerland) site during the period from January 2008 to July 2009. These data were chosen because it was measured in line with the presented natural weathering experiment leaching data (Burkhardt et al. 2012). Potential evapotranspiration was estimated based on temperature, relative humidity, and wind speed using Turc’s formula (Eq. 1) (Turc 1961).

1 $$\text{ETP}=0.0133\cdot \frac{{T}_{m}}{{T}_{m}+15}\cdot \left({R}_{s}+50\right)\cdot C\left[\text{mm}\cdot {d}^{-1}\right]$$

*C* at RH < 50%: $$C=1+\frac{50-\text{RH}}{70}$$
*C* at RH > 50%: *C* = 1
ETP: average pot. Evapotranspiration per day
*T*_*m*_: average monthly air temperature
*R*_*s*_: average global irradiation per day
RH: average monthly relative humidity

### Experimental and simulated emission data from buildings

Biocide emission concentrations were necessary as input parameters for the PELMO. First of all, the soil area affected by the façade runoff needed to be defined. As an orientation the model house described in the emission scenario documents (ESD) for Product type 10 (PT 10): Emission scenarios for biocides used as masonry preservatives (EUBEES 2002) from the ECHA website (2022b)) was used to obtain an appropriate soil/facade ratio. There, a 0.1-m soil layer was described as the width of the test façade used to calculate the surrounding soil area. As the intake parameter, the emission was converted to mg biocide per m^2^ soil surface per timestep.

Three different scenarios were considered in this study, based on the source of input data. In the first scenario (NAT experiment), the natural weathering leaching data previously described by Burkhardt et al. (2012) were used (Table 1).

**Table 1:** Input data for the application scenarios

	NAT	COM_NAT_	COM_DIN_
Weather data	Dübendorf (Switzerland) January 2008 to January 2009	Dübendorf (Switzerland) January 2008 to July 2009	Dübendorf (Switzerland) January 2008 to July 2009
Emission data	TB and OIT: natural weathering experiment Dübendorf (Switzerland) January 2008 to January 2009 ^1^	TB and OIT: natural weathering experiment Dübendorf (Switzerland) January 2008 to January 2009 ^1^ BIT: DIN EN 16105 Immersion test ^2^	TB and OIT: DIN EN 16105 Immersion test ^3^ BIT: DIN EN 16105 Immersion test ^2^
Geometry data	Data derived from Burkhardt et al. (2012)^1^	Model house from the ECHA document ^4^	Model house from the ECHA document ^4^
Material data	From Burkhardt et al. (2012)^1^	From Burkhardt et al. (2012)^1^ and Kiefer et al. (2023)^2^	From Kiefer et al. (2023)^2^ and Schoknecht et al. (2009)^3^
Runoff coefficient	n.a	0.9	0.9

^1^ Leaching data from the natural weathering experiment described by Burkhardt et al. (2012)

^2^ Data from the DIN EN 16105 Immersion test from Kiefer et al. (2023)

^3^ Data from the DIN EN 16105 Immersion test from Schoknecht et al. (2009)

^4^ EUBEES. Emission scenario document for biocides used as masonry preservatives: EUBEES 2002

The biocide concentrations and precipitation volumes were applied to the soil, at limited time points after the rain events. Here the emission values were only available from one artificial façade, oriented to one weather direction. Therefore, the emission values were related to a soil area with a width of 2.5 m (like the artificial facade) and a distance of 0.1 m around the facade.

For the second and the third scenarios, the emission values were generated using the COMLEAM software (OST—Eastern Switzerland University of Applied Sciences, www.comleam.com) (2022a). For the second scenario (COM_NAT_) the emission data from the first scenario (NAT). The third scenario (COM_DIN_) comprised a complete artificial setting where a laboratory DIN leaching test was used to get an emission curve for an upscale, as described by Schoknecht et al. (2009). In-can preservative BIT, which is emitted in the environment rapidly and degrades fast, was not integrated in the previous studies. For simulation, a DIN EN 16105 emission curve described by Kiefer et al. (2023) was used. It needs to be noted that for both COMLEAM simulations, the same leaching data for BIT was used, but different leaching data od OIT and TB.

Except for the data, used to generate the emission curves for the simulation, both scenarios (COM_NAT_ and COM_DIN_) were identical. As an input weather parameter, the weather period of the natural weathering experiment was used. To get the hourly resolved emission values, the scenario was theoretically simulated using the COMLEAM software (2022a). Different emission functions and corresponding mathematical models as well as an experimental validation of the calculated emissions are described in a document from the German Environment Agency (Umweltbundesamt 2022).

For the simulation of the emission, an emission curve (with the data from natural or artificial weathering experiments), building geometry, orientation, material properties, and weather data is necessary. In this study the material properties were held the same as described in the leaching publications, geometry of the model house from the ECHA document was used and as weather data, the weather from the natural weathering experiment in Dübendorf, Switzerland was used corresponding to the period of the actual experiment. A calculation of the wind-driven rain was performed according to European Committee for Standardization 2009 ISO 15927–3:^2009^. The façade runoff coefficient was set to 0.9. The COMLEAM simulations were carried out over a period of 579 days, while the natural weather experiment takes place over 370 days.

### Simulation of biocide distribution in soil using PELMO

For the prediction of soil concentrations of the chosen substances, the Pesticide Leaching MOdel PELMO (FOCUS PELMO version 6.6.1, Fraunhofer IME) was used. PELMO is one of four models originally developed for the risk assessment of plant protection products simulating the leaching to groundwater (FOCUS 2009/2014). In PELMO simulations, the water flow was modeled using a tipping bucket approach with a variable time step, encompassing all hydrological processes (evaporation, transpiration, run-off, precipitation, irrigation). The movement of substances within the soil was simulated using the convection–dispersion equation. Substances considered to degrade via first-order degradation, with rate constants adjusted for depth, soil moisture, and soil temperature. Soil sorption was simulated using the Freundlich equation, and kinetic sorption was considered using the Streck model. Furthermore, the study deviated from standard FOCUS scenarios previously described (Fraunhofer Institute for Molecular Biology and Applied Ecology IME 2022) by applying specific scenarios that describe the leaching of selected biocides in soil. Using the input parameters described in “Study design” and “Experimental determination of adsorption, desorption, and degradation of biocides in the reference soil” sections (Table 2), a simulation experiment was carried out over a period of 579 days for different scenarios. Afterwards, for the COM_DIN_ application scenario simulations were ran with the experimentally determined adsorption and degradation values compared to mean values from the literature.

**Table 2:** Input data for PELMO simulations

	PELMO NAT	PELMO COM_NAT_	PELMO COM_DIN_/EXP	LIT
Weather data	Dübendorf (Switzerland) January 2008 to July 2009	Dübendorf (Switzerland) January 2008 to July 2009	Dübendorf (Switzerland) January 2008 to July 2009	Dübendorf (Switzerland) January 2008 to July 2009
Potential evapotranspiration	Calculated using Turc’s formula	Calculated using Turc’s formula	Calculated using Turc’s formula	Calculated using Turc’s formula
Emission data	NAT	COMNAT	COMDIN	COMDIN
Soil properties	Refesol 02-A	Refesol 02-A	Refesol 02-A	Refesol 02-A
Substance data (degradation and adsorption)	Experimentally determined, see Table 4	Experimentally determined, see Table 4	Experimentally determined, see Table 4	Literature values, Table 4
Soil area (compared to runoff)	Calculated using the model house from the ECHA document ^4^	Calculated using the model house from the ECHA document ^4^	Calculated using the model house from the ECHA document ^4^	Calculated using the model house from the ECHA document ^4^

^4^ EUBEES. Emission scenario document for biocides used as masonry preservatives: EUBEES; 2002

### Statistics

The software origin (OriginPro, Version 2022 OriginLab Corporation, Northampton, MA, USA) was used for the figures, regression analysis, and statistics. Firstly, for the comparison of the application time intervals and concentrations, the normal distribution was assessed with the Shaprio-Wilk test, then a Kruskal–Wallis one-way ANOVA on Ranks followed by a Dunn´s post hoc test. The significance level was set at 95% (*α* = 0.05). For the comparison of the soil distribution, a linear correlation was made and the Pearson correlation coefficient was calculated.

## Results and discussion

### Comparison of the different application scenarios

For all simulations presented in this study weather data from the natural weather experiment previously described by Burkhardt et al. (2012) were used. For generating façade runoff a combination of wind (speed and direction) and precipitation is necessary (Blocken and Carmeliet 2004). In total, the precipitation was at 1032 mm within 297 days, upon 1045 rain events (hourly resolution). The maximum precipitation was 12.8 mm per hour. The average wind direction for the rain periods was 223° and the average wind speed was 1.6 m s^−1^ (Supplement Fig. A2 and Fig. A3).

To estimate the biocide emission from buildings, two COMLEAM simulations were performed for the substances OIT and TB. Since only artificial leaching data, based on the immersion test DIN EN 16105, was available for BIT, the resulting emission curves were used for the following simulations with COMLEAM. In turn, these results served as the basis for the PELMO simulation application scenario. Overall, 1126 mg m^−2^ BIT per soil surface, was applied over the complete period (Supplement Fig. A4). For all biocides, with applications based on COMLEAM (COM_NAT_ and COM_DIN_), simulations were performed with the dataset of 1039 time points with an hourly resolution, whereas for the natural weathering experiment only 34 application time points were provided. The cumulative concentration of the applications in the NAT experiment was 1174 mg m^−2^ for TB and 875 mg m^−2^ for OIT. In the COMLEAM application scenarios, the concentration of the COM_DIN_-based emission was 291 mg m^−2^ for TB and 1241 mg m^−2^ for OIT. The emissions based on the COM_NAT_ simulation were 742 mg m^−2^ for TB and 713 mg m^−2^ for OIT. The application time points of both COM simulations differ significantly from the NAT experiment (*p*-values < 0.0001). Considering only the application concentration, the COM simulated application concentrations were summed up over the sampling period corresponding to the NAT experiment, and the results shifted. Applications of OIT show no significant difference, but in TB, the COM_DIN_ scenarios vary from both COM_NAT_ and NAT simulation (*p*-value < 0.0001).

**Fig. 2:**
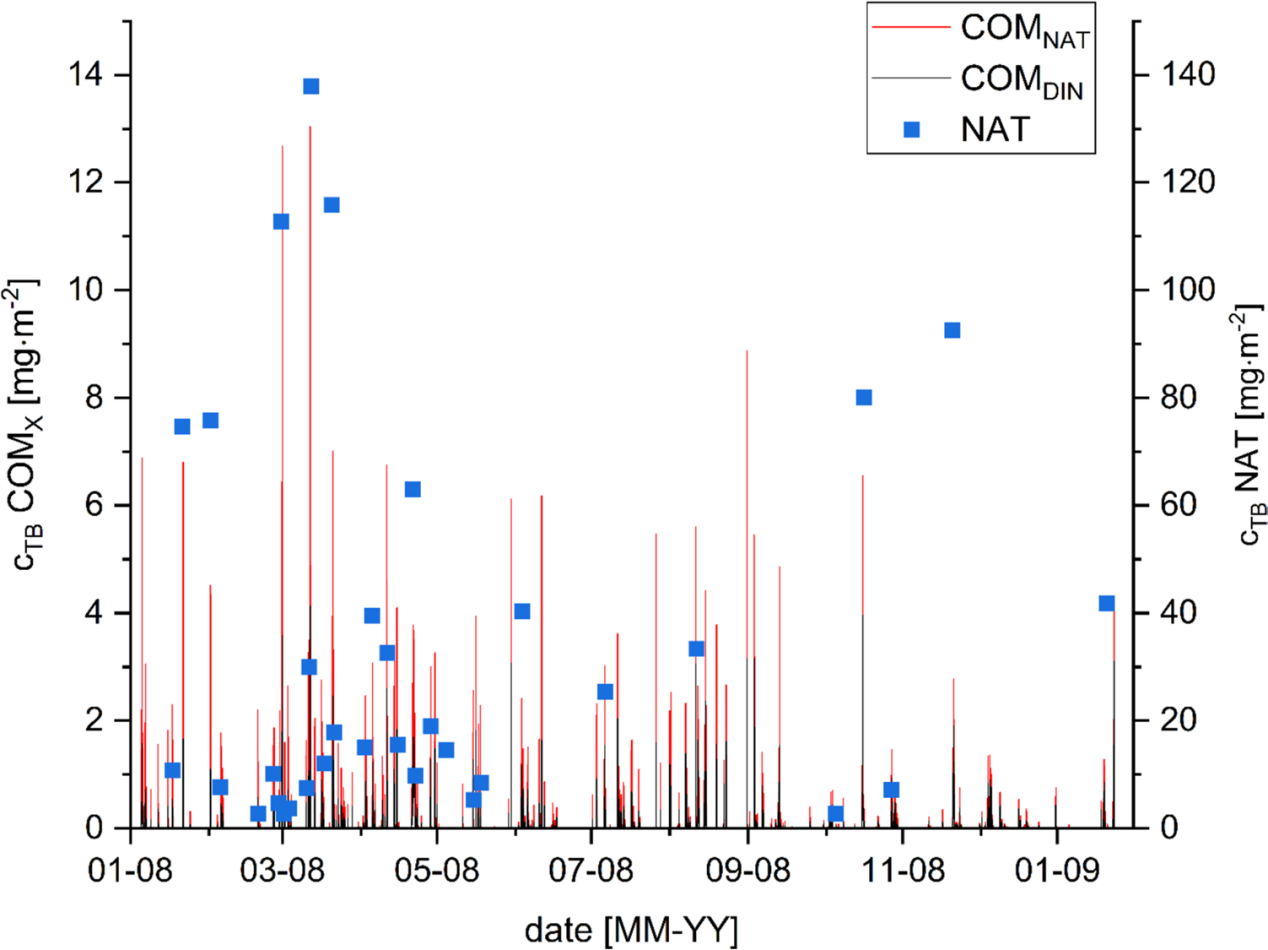
Emission concentrations of TB. The black bars show the application concentrations based on the DIN EN 161005 emission function simulated with COMLEAM software and the red bars show the application concentrations based on the natural weather (NAT) experiment as an emission function for the simulation with the COMLEAM software, the values can be seen at the left scale. The blue data points show the application concentration of the natural weather experiment without further modifications, the values can be seen at the right scale

Within the COM simulations, every rain event causes a runoff, even if the biocide concentrations are low. The peak concentrations of 4.73 mg m^−2^ for COM_DIN_ and 13.04 mg m^−2^ for COM_NAT_ were reached in the case of TB application. During the NAT experiment, as compared to the COMLEAM simulations, higher concentrations of TB were applied with peak concentrations around 137.88 mg m^−2^, but with significantly fewer overall application timepoints, followed by longer periods of rainfall (Fig. 2). In the case of OIT, the overall biocide amount was at its highest for COM_DIN_, with peak concentrations of 18.86 mg m^−2^ for COM_DIN_ and 14.65 mg m^−2^ for COM_NAT_. Whereas in the course of the NAT, experiment peak concentrations of 150.00 mg m^−2^ were detected in the runoff applied to PELMO (Supplement Fig. A5, the cumulative emission of all biocides and scenarios is shown in Supplement Table A2).

In the NAT and COM_NAT_ experiments, the highest cumulative amount of TB, but a lower cumulative amount of OIT, compared with COM_DIN_ was detected. The combination of the natural weathering experiment with COMLEAM resulted in an amount of TB average of the three scenarios and predicted the lowest concentration for OIT. In contrast, COM_DIN_ shows the highest application values for OIT and the lowest for TB.

The differences between the application concentrations for the three different scenarios can be explained by the nature of the weathering experiments. The advantage of a natural weathering experiment is that all weather parameters like UV irradiation, temperature changes, frost, dew, and others are covered. The major disadvantage of such experiments is reproducibility, rendering data not comparable amongst different locations and dates. (Reiß et al. 2021) All weather parameters were directly included for the NAT experiment and indirectly used for the experiment coupled with COMLEAM because weather parameters influence the emission curve. The degradation of biocides, e.g., through UV radiation, is indirectly considered via COMLEAM in combination with a natural weather experiment, in which the emission curve data contains lower concentrations if biocides have degraded on the façade. Active consideration of degradation rates of individual biocides is not possible with a COMLEAM simulation. In the third and last scenario, the artificial DIN EN 16105 leaching experiments (Schoknecht et al. 2009) were used to generate leaching data for the following COMLEAM simulation emission curve. The major disadvantage of artificial weathering is that weather parameters, e.g., UV-irradiation, temperature changes, frost, dew, and others are not anticipated. Additionally, herein the water façade ratio is very high, which leads to an overestimation of the leaching potential. On the other hand, the advantages are its simple, time-efficient methodology with good reproducibility and comparability (Schoknecht et al. 2013).

In both COM_DIN_ and COM_NAT_ scenarios, the weather data, the geometry, and the material applied can be defined and the application of different biocides in varying concentrations can be adapted easily. The emission curves for every biocide and material are necessary to perform the simulation (Eq. 2 (emission function for the leaching simulation with COMLEAM) and Table 3).

**Table 3:** Emission curve parameters for the different simulation scenarios

Experimental data	a [mg m^−2^]	b [m^2^ L^−1^]
BIT [DIN EN 16105, ^1^]	151.315	1.79
OIT [DIN EN 16105, ^2^]	346.577	0.013
TB [DIN EN 16105, ^2^]	121.201	0.008
OIT [Natural weathering, ^3^]	16.023	0.515
TB [Natural weathering,^3^]	33.896	0.135

^1^ Data from the DIN EN 16105 Immersion test from Kiefer et al. (2023), ^2^ data from the DIN EN 16105 Immersion test from Schoknecht et al. (2009), ^3^ data from the natural weathering experimentBurkhardt et al. (2012)

2 $${E}_{cum}=a\cdot ln\left(1+b\cdot {q}_{c,cum}\right)$$

The soil application frequency differs greatly when data from the NAT experiment was used as an input parameter for PELMO simulations directly, from that with a dataset further processed with COMLEAM (COM_NAT_). For the natural weathering experiment only 34 application points with higher biocide concentrations were applied, compared to the COMLEAM simulations, leading to shorter biocide presence times in soil.

Comparing only the COMLEAM simulated application scenarios, the emission data and the generated emission curve caused the difference in the application concentrations between the scenarios. Due to the influence of all weathering parameters on the natural weather emission curve, the amount of the more hydrophobic TB in the PELMO application was still slightly higher than in OIT. For the DIN emission curve, higher concentrations of OIT were predicted. In general, the simulation of the DIN 16105 in combination with COMLEAM (COM_DIN_) resulted in the lowest values of TB and the highest values of OIT. All COMLEAM simulations have a significant impact on the application frequency compared to natural weathering experiment (NAT). In terms of application concentrations, only TB resulted in a significant difference between the NAT experiment, and corresponding emission curves, compared to COM_DIN_ for further simulations.

Comparing DIN-based emission curves to NAT experiment-based emission curves led to significantly different results for more hydrophobic compounds, while more hydrophilic biocide results are comparable. This conclusion only stands true for the leaching process and not for the soil distribution.

### Distribution of biocides in soil using PELMO

#### Influence of different application scenarios for further PELMO simulations

**Fig. 3:**
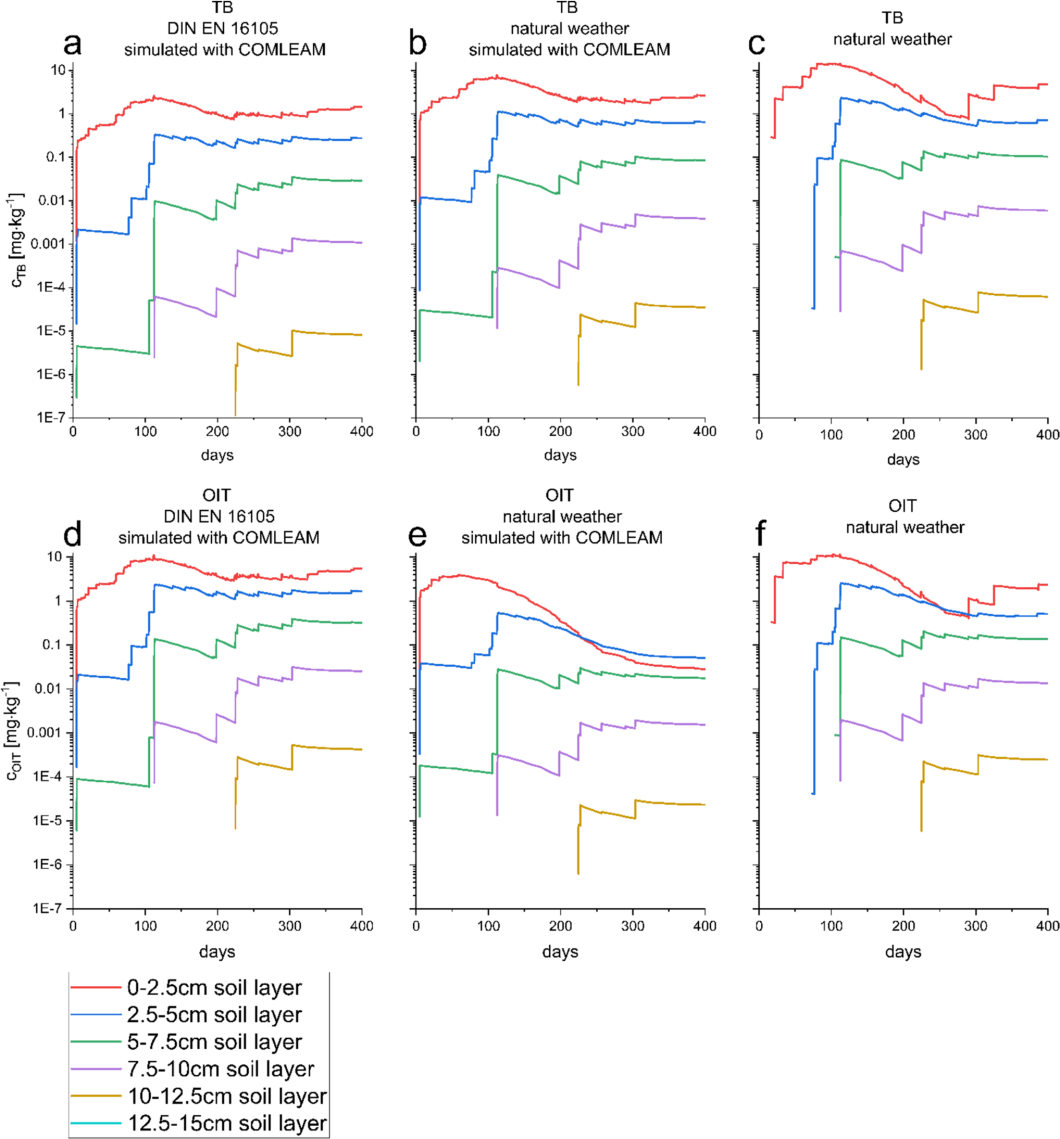
Distribution of TB (**A**, **B**, **C**) and of OIT (**D**, **E**, **F**) after PELMO simulation in five different soil layers, 0–2.5 cm (red), 2.5–5 cm (blue), 5–7.5 cm (green), 7.5–10 cm (violet), and 10–12.5 cm (orange). Graph **A** depicts the distribution based on the application concentration, simulated using COMLEAM and DIN EN 16105-based leaching data, represented as an emission curve. Graph **B** illustrates the distribution based on the application concentration observed in the natural weather experiment, without additional simulations. Graph **C** shows the distribution based on the application concentration, simulated using COMLEAM and incorporating the leaching data from the natural weather experiment as an emission curve

To investigate how the differences amongst scenarios described above (NAT, COM_NAT_, and COM_DIN_) affect the biocide distribution in different soil layers a PELMO simulation with the three scenarios were performed on five soil layers (0–2.5 cm, 2.5–5 cm, 5–7.5 cm, 7.5–10 cm, and 10–12.5 cm) (Fig. 3).

The soil concentrations of the different application scenarios were compared at a single time point (300 days). In the case of TB, all scenarios just differed within one order of magnitude regardless of the layer. The concentrations of TB in the upper layer 0–2.5 cm in the first scenario (NAT) was 2.54 mg kg^−1^, in the second scenario (COM_NAT_) 1.96 mg kg^−1^, and 0.95 mg kg^−1^ in the third (COM_DIN_), with the difference of the intake scenarios in a range of 1.8 mg kg^−1^ ± 39%. For deeper soil layers (7.5–10 cm), this influence of the application scenarios was shown to be less dominant. The corresponding concentrations were 0.10 mg kg^−1^ for the NAT, 0.073 mg kg^−1^ for the COM_NAT_, and 0.024 mg kg^−1^ for the COM_DIN_ scenarios. In this layer, the concentration ranged around 0.066 mg kg^−1^ ± 4.6%. The deeper soil layer concentrations differ by at least one order of magnitude. However, the variation of the values decreases for the two presented values from 39 to 5%. For a better comparison with all time points a linear correlation between the scenarios for every layer was conducted. In the case of TB, a linear correlation (Pearson’s *R* above 0.589) can be observed for all layers and scenarios (Supplement Table A3). Additionally, the strength of the correlation increases with higher soil depth (Pearson’s *R* above 0.855 at and above layer 5–7.5 cm).

In the case of OIT, a correlation between the scenarios could be observed too (Pearson’s *R* above 0.511) with an exception of the two COMLEAM-based (COM_NAT_ and COM_DIN_) scenarios. Here, for the first two soil layers, the *R*-value was below 0.5. For the 0–2.5 cm layer, almost no correlation could be observed (Pearson’s *R* = 0.065), and for the 2.5–5 cm layer a weak correlation could be demonstrated (Pearson’s *R* = 0.363). Nevertheless, the same trend as that of TB was observed, in the sense that the correlation between all three scenarios becomes stronger in deeper soil layers (Pearson’s *R* is ≥ 0.576 starting with 5–7.5 cm depth).

The long half-life of TB is potentially a high risk to the soil environment. Consequently, it has already been banned since 2004 for use as a pesticide (Quednow and Püttmann 2007). Because of the already indicated high risk for the soil environment, the influence of the façade eluates should be considered as another risk factor, e.g., soil infiltration within urban regions. In the case of OIT, the COM_DIN_ concentrations overestimated the biocide concentration in the upper soil layer. If this scenario is employed for risk assessment, it should be carefully interpreted and taken as the worst-case scenario.

As a model of hydrophilic and fast degradable in-can preservative, BIT was used in this study., Herein the concentrations in soil were detected to decrease rapidly, and the maximum concentration in the 0–2.5 cm soil layer was 4.11 mg kg^−1^ on day 6 and 1.56 mg kg^−1^ on day 113. The concentrations of BIT decreased with the soil depth, peak concentrations were detected on day 113 reaching 0.07 mg kg^−1^ at 2.5–5 cm layer and 0.00087 mg kg^−1^ at 5–7.5 cm depth (Supplement Fig. A6).

The half-life of biocides in soil depends on many parameters, e.g., soil properties, temperature, microbiome, soil humidity. There is a possibility that biocides may still be present in the soil after many years. With a combination of PELMO and COMLEAM, the extension of the simulation to longer time periods is necessary for a risk assessment over time periods that cannot be covered by field studies. The estimation of the long-time effect, accumulation tendencies, and soil depth distributions, the combination of different leaching experiments with COMLEAM and PELMO is now offering a powerful tool, with easy adaptability to different building geometries, material properties, and weather parameters.

#### Influence of adsorption and degradation values using PELMO to simulate the distribution of biocides from facades in soil

Degradation and adsorption were reported to be important parameters for modeling the behavior of biocides and pesticides in soil (Dubus et al. 2003; Vega-Garcia et al. 2022). To evaluate the influence of these parameters, both were experimentally determined for the reference soil and compared with mean values for the corresponding biocides as to establish whether the determination of the compounds is necessary to simulate the behavior of biocides in soil. However, the experimental determination of these soil-dependent values for all substances is costly and time-consuming. A simpler approach could be to use literature values, but these are usually mean values or values obtained with different soils. Literature values and experimentally determined values were compared to determine if the cheaper and faster approach using literature values could be beneficial.

The soil adsorption parameter K_OC_ was determined according to the OECD 106 (OECD and Organisation for Economic Co-operation and Development 2000). BIT showed the strongest adsorption tendency (K_OC_ 2110.9 mg L^−1^), and was the only biocide with reversible adsorption. TB had a K_OC_ of 1206.0 mg L^−1^ and OIT had the weakest adsorption tendency out of the described biocides with K_OC_ of 812.2 mg L^−1^. Experimentally determined degradation values show that BIT is rapidly degradable with half-life below one day, whereas OIT and TB were much more persistent with determined half-lives of 14 days.

The literature values the half-lives of BIT confirm the findings above, but the adsorption of BIT was reported to be much weaker with a K_OC_ value of 196.87 L kg^−1^ (ECHA 2021). For OIT the assessment report of product type 8 (wood preservative) was used as an information source. Herein, the degradation value, DT_50_ of 0.9 days for aerobic soil biodegradation, adsorption value, K_OC_ of 982 L kg^−1^, and a 1/*n* = 0.8427 were reported as the geometric mean of three soils and one sediment (ECHA 2021). Therefore, literature data on OIT provide an adsorption value close to that experimentally determined, but a shorter half-life value. No assessment reports are available for TB, instead, the Pesticide Properties DataBase (PPDB) reported by Lewis et al. (2016) was used as an information source. The degradation, DT_50_ values of 74 days (typical), 74 days (lab at 20 °C), and 52 days (field) are reported. As adsorption parameters, herein, K_oc_ values 518 (range EU dossier (*n*_soil_ 5: 392–605 mL g^−1^) and 1/*n* values = 1.10 (range EU dossier (*n*_soil_ 5: 1.01–1.35) were provided (University of Hertfordshire 2022) (Table 4).

**Table 4:** Substance-specific properties for the biocides BIT, TB, and OIT

	BIT		OIT		TB	
Parameter	Experimental	Literature^1^	Experimental	Literature^2^	Experimental	Literature^3^
DT_50_ [days]	1	1	14	0.9	14	74
K_OC_ [L kg^−1^]	2110.7	196.87	815.2	982	1206	518
Freundlich exponent	n.a	n.a	0.9	0.8427	0.9	1.1

^1^ The literature values for BIT were used based on the report “Opinion on the application for approval of the active substance: 1,2 BENZISOTHIAZOL-3-(2H)-ONE (BIT)” (ECHA 2021). ^2^ The literature values for OIT were used based on the assessment report as product type 8 (wood preservative) (ECHA 2021). ^3^ The literature values for TB were based on the Pesticide Properties DataBase (PPDB) (University of Hertfordshire 2022)

The adsorption and degradation values in the literature vary, not only due to strong dependence on soil properties such as carbon content, but also because of the experimental condition variability (i.e. microbial active soil, temperature, humidity). Nevertheless, a general trend can be derived, since both the in-house determined values and the literature values of BIT, a polar in-can preservative, are shown to be rapidly degradable (DT_50_ < one day). OIT as a model of moderate hydrophobic film preservatives, demonstrates high variations in the degradation time (14 days experimental determined and < 1 day in the literature) but has comparable K_OC_ values. In the case of, TB, as an example of the most persistent and hydrophobic biocide, the half-lives reported in the literature (DT_50_ 74 days) exceed that of evaluated (DT_50_ 14 days) and vary greatly amongst sources from 9 up to 231 days. (Bollmann et al. 2017; Daho 2006; Donati and Funari 1993; Lewis et al. 2016).

The wide ranges for degradation and adsorption values found in the literature and findings herein demonstrate the impact of the soil properties on these parameters. Vega-Garcia et al. (2022) demonstrated simulations to estimate the adsorption and degradation of the various biocide transformation products but indicated that experimental values are needed to validate the simulations. Furthermore, temperature and therefore microbial activity could influence the biocide adsorption and degradation in different soil layers and should be taken into account.

#### Adsorption and degradation influence on the predicted biocide concentration in soil

In comparing the results of the PELMO simulation based on the experimentally determined values (EXP) with the literature value-based simulation (LIT), the curves for each layer show a linear correlation between EXP and LIT values (Supplement Table A4). Pearson’s *R* was always found above 0.904 for BIT and 0.729 for TB, which indicates that the curve trends were comparable, whereas the OIT Pearson *R* indicates a correlation only for the 0–2.5 cm layer with an *R*-value of 0.604, and the *R*-value was below 0.5 for the deeper layers. Compared to the correlation of the different application scenarios (NAT, COM_NAT_, and COM_DIN_) described above, where the biocide concentrations range mostly within one order of magnitude for corresponding soil layers, the predicted values for varying adsorption and degradation are much higher, especially for the deeper soil layers.

The predicted TB soil concentration (0–2.5 cm layer) was 7.78 mg kg^−1^ in the EXP simulation and 9.34 mg kg^−1^ in the LIT simulation. The lowest predicted concentrations were 0.0053 mg kg^−1^ in both cases. Furthermore, the highest and lowest values were predicted to occur at the same time points for both simulations. For the deeper soil layers, the value variation was shown to increase. For example, at the 7.5–10 cm soil layer, a peak TB concentration of 4.90 × 10^−3^ mg kg^−1^ and 1.18 × 10^−5^ mg kg^−1^ as the lowest value was predicted by the EXP simulation. The LIT simulation predicted the highest soil concentration of 1.15 mg kg^−1^ and the lowest concentration of 0.00076 mg kg^−1^. The minimal biocide concentration was predicted to occur simultaneously for both simulations, on the date the biocide reached a deeper soil layer. The highest values varied, predicting day 303 via the EXP simulation, and day 564 via LIT simulation. This trend was visible for all soil layers deeper than 2.5 cm (Supplement Table A5). The trend of the maximum predicted concentration occurring at the end of the simulations in the LIT simulation reflected the increased accumulation tendency of TB due to the longer half-lives. Herein the literature value of a DT_50_ of 74 days was chosen for the degradation of TB.

For rapidly degradable biocides like BIT with half-lives ranging within one day, the predicted soil concentrations of the upper soil layer (0–2.5 cm) were comparable, with concentrations of 4.09 mg kg^−1^ via the EXP simulation and 4.01 mg kg^−1^ via the LIT simulation. However, the time points of the predicted peak concentration occurrence differ. According to the EXP simulation, the highest concentration is predicted at day 60, and day 71 for the LIT simulation. The lowest predicted values were 0.0026 mg kg^−1^ in the EXP and 0.0042 mg kg^−1^ in the LIT simulation, both predicted to occur on day 182. For the deeper soil layers, the difference in the predicted values increased. For the soil layer 7.5–10 cm, the EXP simulation predicted maximum concentrations of 2.19 × 10^−6^ mg kg^−1^ and lowest concentration of 6.73 × 10^−13^ mg kg^−1^. Peak concentrations of 0.0079 mg kg^−1^ and lowest concentrations of 2.43 × 10^−9^ were predicted via LIT. Both events occurred within the same week, the maximum concentration for both was predicted on day 113 and the lowest predicted concentrations were predicted after 199 days and 194 days via the EXP and LIT simulation, respectively. In the case of BIT, the half-lives of the LIT and EXP simulations were comparable but the adsorption values differed, as opposed to findings for TB where the adsorption values were significantly more comparable than the half-lives. It leads to reason that the accumulation tendency of biocides in soil is dominantly affected by their half-lives, while the adsorption mainly dictates the peak concentrations of biocides in soil layers.

The adsorption and degradation values used in the EXP and LIT simulations (Table 2) for OIT differed considerably. With an experimentally determined DT_50_ value of 14 days, OIT exhibits median persistent properties like those observed in the case of TB. According to the literature, the DT_50_ value is less than one day, as a result, OIT shows a behavior of rapidly degradable biocides such as BIT via LIT simulation. OIT is the only addressed biocide where the changing of the adsorption and degradation values influences not only the predicted concentrations but also the course of the predicted concentration over time. Nevertheless, the predicted concentrations for the upper soil layers (0–2.5 cm) were comparable, with maximum concentrations of 3.94 mg kg^−1^ and 1.60 mg kg^−1^ and minimum concentrations of 0.000057 mg kg^−1^ and 4.44 × 10^−20^ mg kg^−1^ via the EXP and the LIT simulations, respectively. The occurrence of the event reaching the maximum values was found to be day 21 for both simulations. The lowest concentration was predicted to occur after 354 days, which reflects the trend of the medium fit for the upper layer detected for OIT (Pearson *R* 0.604). For deeper soil layers (7.5–10 cm), the simulated concentrations varied from 0.0020 mg kg^−1^ to 0.000015 mg kg^−1^ for the EXP and from 5.12 × 10^−10^ mg kg^−1^ to 4.34 × 10^−20^ mg kg^−1^ for the LIT simulation (Supplement Table A5).

These findings demonstrate that a high variability in the degradation time, like in the case of OIT, influences the course of the distribution curves: In contrast, a difference in the adsorption, like in the case of BIT, does not affect the course but rather influences the predicted concentrations.

**Fig. 4:**
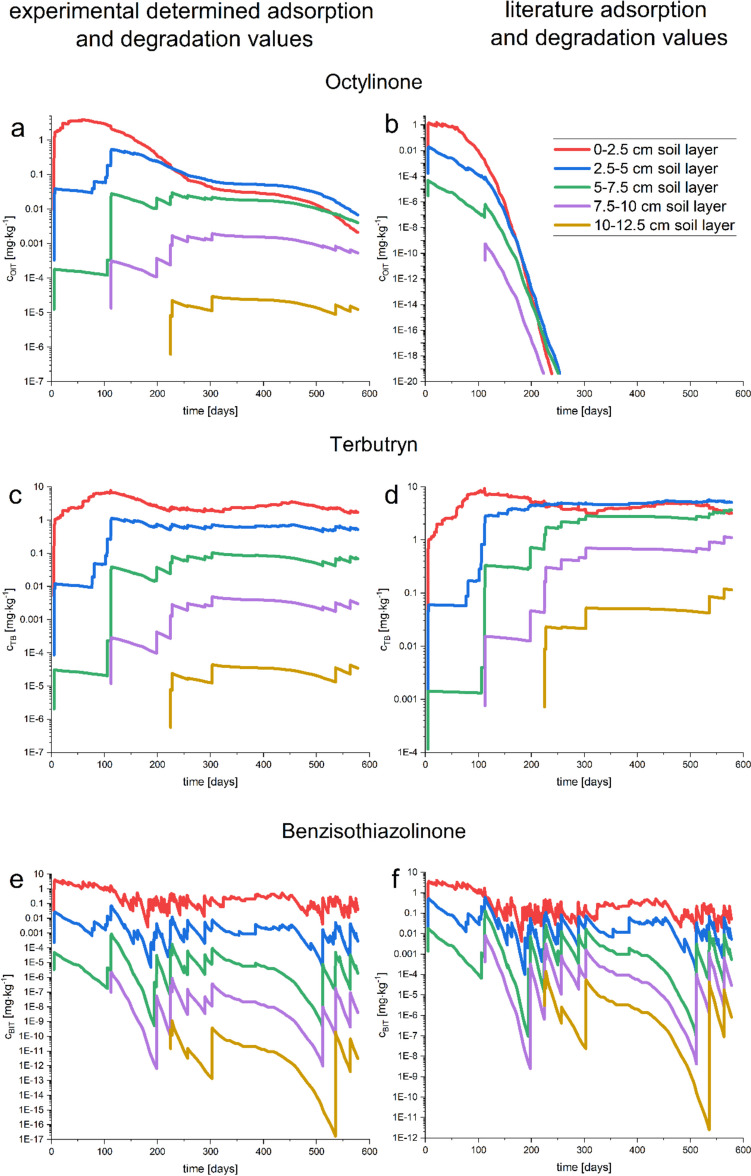
Simulated distribution of OIT (**A**, **B**), TB (**C**, **D**), and BIT (**E**, **F**) in soil. The predicted concentration in different soil layers using the experimentally determined adsorption and degradation values (**A**, **C**, **E**). The simulated results using adsorption and degradation values from the literature (**B**, **D**, **F**). At the top, the OIT distribution is shown, in the middle—TB, and at the bottom—BIT

For rapidly degradable biocides such as BIT only a short-term risk for the environment exists, even leached out over a time period of 1.5 years, since they are degraded rapidly upon the last intake. Furthermore, previous leaching studies showed that BIT leaches out at the beginning of a façade lifetime, mostly in the first weeks, or during the first wetting events (Kiefer et al. 2023; Styszko et al. 2015). In comparison to film preservatives, which have been found continually in low concentrations in wastewater treatment plants, in-can preservatives such as BIT were found in peak concentrations at a few events (Bollmann et al. 2014). On the other hand, such biocides are found in high concentration and thus should not be ignored, particularly considering short-term risks for the environment. For more persistent biocides such as TB, long-term risks are already predictable because of the increasing concentration in deeper soil layers over time (Fig. 4). Since film preservatives leach out over a long period of time, the application time could exceed the simulated 600 days, which is even more concerning for soils with a slower degradation impact against TB. Furthermore, the DT_50_ value determined in-house experimentally (14 days) was much faster than that reported in most studies (Bollmann et al. 2017; Lewis et al. 2016). Considering lower degradation rates for TB, with a DT_50_ value of 74 days and higher, TB might be accumulating in the soil. According to the literature DT_50_ values, the concentration in the deeper soil layers exceeded the concentration in the upper layer and showed a clear accumulation tendency (Fig. 4).

Biocide-specific values such as adsorption and degradation are important for an estimation of the environmental risk and should be determined experimentally. Amongst the soil parameters, at the very least organic content, pH, and where applicable,microbial activity should be known. In general, properties of the soil, such as texture, organic matter content, pH, and moisture levels, significantly affect the behavior and fate of substances. The two most important parameters are organic carbon content and the soil texture. The soil texture, determined by the proportions of sand, silt, and clay, controls water retention, permeability, and porosity. Fine-textured soils (e.g., clay) retain substances more effectively due to smaller pore spaces and greater surface area, reducing vertical mobility. Coarser soils (e.g., sandy) enhance leaching due to higher permeability. Organic carbon influences the environmental fate of substances by controlling adsorption, mobility, and degradation dynamics, e.g., high OC content in humus-rich soils results in greater adsorption, less mobility, and slower degradation for certain substances due to reduced bioavailability. The substance-specific parameters for adsorption (Koc) and DT50 are universally sensitive parameters, as their values dictate the retention and persistence of the substances. As is already established in the case of pesticides in agricultural use, both degradation and adsorption decrease in deeper soil layers, due to changes in soil carbon content, temperature, and microbial activity (Fierer 2017; Jenks et al. 1998; Larsbo et al. 2009; Pantelelis et al. 2006; Reedich et al. 2017; Sonia Rodríguez-Cruz et al. 2006). These effects should further be confirmed for the façade scenario, in line with the above-mentioned parameters, within different soil layers should be evaluated experimentally, to cover the complex soil properties, and thus changing biocide behavior field studies are necessary for the validation of this approach. Additionally, structural identification and knowledge about the behavior of biocidal degradation products, e.g., their persistence in different environments, and their ecotoxicological potential, are factors that need to be considered in further studies and integrated in the simulation model.

## Conclusion

Simulating the behavior of biocides in soil by combining the software tools COMLEAM, developed for facade leaching, and PELMO, developed for pesticide leaching, leads to results that may overestimate the risk of short-acting in-can preservatives such as BIT, but appear adequate for selected film preservatives. Key parameters for prediction are adsorption and degradation of biocides, which are strongly dependent on soil properties. The predicted concentrations differ less in the upper soil layer, but the difference increases with soil depth. The data should therefore be determined experimentally or selected on a well-founded basis. The input scenarios (hourly resolution) showed only a small effect on the upper soil layers, but this difference did not exceed one order of magnitude. This makes it possible to use simple and reproducible test procedures, such as the DIN 16105 immersion test, to generate the emission curves for the simulation with COMLEAM with sufficient accuracy. Since COMLEAM can be easily adapted to different geometries, buildings, weather, and material parameters, the software in combination with PELMO is a suitable tool for predicting leaching and estimating soil contaminations.

## Supplementary Information

Below is the link to the electronic supplementary material.

Supplementary file1 (DOCX 131898 KB)

## Data Availability

The authors state that the data supporting the study’s findings are included in the paper and in the supplementary information files. If raw data in another format or presentation is required, it can be obtained from the corresponding author upon request.
