# Supplementary material for: Simulation of the biocide distribution in soil using PELMO coupled with COMLEAM
Source: Environ Sci Pollut Res Int. 2025 Jan 9;32(5):2425–40. doi: 10.1007/s11356-024-35760-y (PMC11802628; doi:10.1007/s11356-024-35760-y)
Supplement: Supplementary file 1 — Supplementary file1 (DOCX 131898 KB) [file 11356_2024_35760_MOESM1_ESM.docx]

*Figure A1:* *Flowchart of the five investigated simulation scenarios carried out with PELMO for the time-resolved prediction of the concentrations of biocides OIT, terbutryn and BIT in the respective soil depths*

*The first column of the flow chart shows the PELMO NAT simulation, which was simulated on the basis of a natural weather experiment. The second column of the flow chart shows the PELMO simulation based on the COM_NAT_ input data. In this case, a COMLEAM simulation based on the natural weather experiment was performed before the PELMO simulation. The third column of the flow chart shows the PELMO simulation with the COM_DIN_ input scenario, which is based on a laboratory leaching experiment. Furthermore, experimental or literature data for adsorption and degradation were used for the PELMO simulation.*

*^1^ leaching data from the natural weathering experiment described by Burkhardt et al. (2012)*

^2^ weather data from the natural weathering experiment period described by Burkhardt et al. (2012)

^3^ data from the DIN EN 16105 Immersion test from Schoknecht et al. (2009)

^4^ data from the DIN EN 16105 Immersion test from Kiefer et al. (2023).

Table A1: Information about the soil RefeSoil02A

| Parameter | value |
| --- | --- |
| Sand content [%] | 1.7 |
| Silt content [%] | 82.6 |
| Clay content [%] | 15.6 |
| C_org_ | 1.06 |
| pH [H_2_O] | 7.44 |
| Field capacity [m³·m^-^³] | 0.4278 |
| Withering point [m³·m^-^³] | 0.1052 |
| Dispersion length [cm] | 5 |

Figure A2: Weather parameters for the location of Dübendorf, Switzerland are shown. The rain amount over the observed time period (blue bars) as well as the global radiation (grey bars) and the temperature (red bars) are given.

Figure A3: Wind direction and speed for Dübendorf, Switzerland over the observed time period is given in figure A. Figure B shows the wind direction and speed only for the times where the precipitation is ≠0.

*Figure A4: Emission concentrations of BIT. The black stripes show the application concentrations based on the DIN EN 16105 emission function simulated with COMLEAM software.*

Figure A5: Emission concentrations of OIT. The black stripes show the application concentrations based on the DIN EN 161005 emission function simulated with COMLEAM software and the red stripes show the application concentrations based on the natural weather experiment as emission function for the simulation with the COMELAM software, the values can be seen at the left scale. The red data points show the application concentration of the natural weather experiment without further modifications, the values can be seen at the right scale.

Table A2: Cumulative application concentration of the different biocides for the different application scenarios

|  | **BIT [mg·m^-2^]** | **OIT [mg·m^-2^]** | **TB [mg·m^-2^]** |
| --- | --- | --- | --- |
| **NAT** | n.a. | 875 | 1174 |
| **COM_NAT_** | 1126 | 713 | 742 |
| **COM_DIN_** | 1126 | 1241 | 291 |

Figure A6: The figure gives the distribution of BIT simulated with PELMO for five different soil layers, 0 – 2.5 cm (red line), 2.5 – 5 cm (blue line), 5 – 7.5 cm (green line), 7.5 – 10 cm (violet line), and 10 – 12.5 cm (orange line)

Table A3: The Pearson R-values. They were calculated based on a linear correlation between two application scenarios for each soil layer.

| **Comparison TB (Pearsons´R)** | | | |
| --- | --- | --- | --- |
| soil layer | DIN (COMLEAM) - natural weather (COMLEAM) | natural weather - DIN (COMLEAM) | natural weather - natural weather(COMLEAM) |
| 0-2.5 cm layer | 0.755 | 0.616 | 0.953 |
| 2.5 -5 cm layer | 0.911 | 0.589 | 0.859 |
| 5 - 7.5 cm layer | 0.981 | 0.855 | 0.934 |
| 7.5 - 10 cm layer | 0.992 | 0.958 | 0.985 |
| 10 - 12.5 cm layer | 0.993 | 0.966 | 0.989 |
| **Comparison OIT (Pearsons´R)** | | | |
| soil layer | DIN (COMLEAM) - natural weather (COMLEAM) | natural weather - DIN (COMLEAM) | natural weather - natural weather(COMLEAM) |
| 0-2.5 cm layer | 0.065 | 0.512 | 0.815 |
| 2.5 -5 cm layer | 0.363 | 0.574 | 0.966 |
| 5 - 7.5 cm layer | 0.577 | 0.775 | 0.958 |
| 7.5 - 10 cm layer | 0.917 | 0.947 | 0.996 |
| 10 - 12.5 cm layer | 0.941 | 0.960 | 0.998 |

Table A4: The Pearson R-values for the comparison between literature values-based simulation and experimental values-based simulation. They were calculated based on a linear correlation between the same application scenario for each soil layer.

| **Comparison Pearsons´R** | | | |
| --- | --- | --- | --- |
| soil layer | OIT | BIT | TB |
| 0cm [application layer] | 0.948 | 0.683 | 0.902 |
| 0-2.5 cm layer | 0.604 | 0.988 | 0.869 |
| 2.5 -5 cm layer | -0.147 | 0.904 | 0.729 |
| 5 - 7.5 cm layer | -0.413 | 0.991 | 0.902 |
| 7.5 - 10 cm layer | -0.133 | 0.998 | 0.897 |
| 10 - 12.5 cm layer | - | - | 0.885 |

Table A5: Maximum and minimum concentrations for each soil layer over the simulated time period and the appearing days thereof for the comparison between the literature-based simulation compared to the simulated based on the experimental determined values.

| **Maximum peak concentration** | | | | | | |
| --- | --- | --- | --- | --- | --- | --- |
| soil layer [cm] | OIT [mg·kg^-1^] | | TB [mg·kg^-1^] | | BIT [mg·kg^-1^] | |
|  | experimental | literature | experimental | literature | experimental | literature |
| 0 [application layer] | 12.03 | 12.17 | 32.95 | 28.94 | 42.35 | 31.20 |
| 0 - 2.5 | 3.94 | 1.60 | 7.78 | 9.34 | 4.091 | 4.01 |
| 2.5 5.0 | 0.54 | 0.02 | 1.15 | 5.73 | 0.07 | 0.59 |
| 5.0 - 7.5 | 0.03 | 4.70E-05 | 0.10 | 3.71 | 8.60E-04 | 0.13 |
| 7.5 - 10.0 | 0.002 | 5.12E-10 | 4.90E-03 | 1.15 | 2.19E-06 | 7.90E-03 |
| 10.0 - 12.5 | 3.00E-05 | 0.00E+00 | 4.41E-05 | 0.12 | 1.12E-09 | 1.40E-04 |
| soil layer [cm] | OIT [day] | | TB [day] | | BIT [day] | |
|  | experimental | literature | experimental | literature | experimental | literature |
| 0 [application layer] | 22 | 21 | 80 | 80 | 59 | 71 |
| 0 - 2.5 | 60 | 21 | 112 | 112 | 7 | 182 |
| 2.5 5.0 | 114 | 7 | 114 | 535 | 113 | 113 |
| 5.0 - 7.5 | 228 | 5 | 303 | 574 | 113 | 113 |
| 7.5 - 10.0 | 304 | 113 | 303 | 564 | 113 | 113 |
| 10.0 - 12.5 | 304 |  | 303 | 564 | 228 | 228 |
| **Minimum peak concentrations** | | | | | | |
| soil layer [cm] | OIT [mg·kg^-1^] | | TB [mg·kg^-1^] | | BIT [mg·kg^-1^] | |
|  | experimental | literature | experimental | literature | experimental | literature |
| 0 [application layer] | 0.000057 | 4.44E-20 | 0.13 | 0.023 | 0.00032 | 0.00 |
| 0 - 2.5 | 0.00095 | 4.16E-20 | 0.0053 | 0.0053 | 0.0026 | 0.0042 |
| 2.5 5.0 | 0.00 | 4.12E-20 | 0.000086 | 0.00075 | 4.85E-06 | 0.0001 |
| 5.0 - 7.5 | 0.000012 | 4.35E-20 | 0.00 | 0.00011 | 5.35E-10 | 9.44E-08 |
| 7.5 - 10.0 | 0.000015 | 4.34E-20 | 1.18E-05 | 0.00076 | 6.73E-13 | 2.43E-09 |
| 10.0 - 12.5 | 6.23E-07 |  | 5.69E-07 | 0.00 | 1.67E-17 | 1.68E-12 |
| soil layer [cm] | OIT [day] | | TB [day] | | BIT [day] | |
|  | experimental | literature | experimental | literature | experimental | literature |
| 0 [application layer] | 654 | 354 | 5 | 384 | 182 | 182 |
| 0 - 2.5 | 5 | 239 | 5 | 5 | 182 | 182 |
| 2.5 5.0 | 5 | 254 | 5 | 5 | 188 | 188 |
| 5.0 - 7.5 | 5 | 251 | 5 | 5 | 194 | 198 |
| 7.5 - 10.0 | 113 | 223 | 112 | 113 | 198 | 194 |
| 10.0 - 12.5 | 225 |  | 224.00 | 225.00 | 536.00 | 536.00 |

References

Burkhardt, M.; Zuleeg, S.; Vonbank, R.; Bester, K.; Carmeliet, J.; Boller, M.; Wangler, T. (2012): Leaching of biocides from façades under natural weather conditions. In: *Environmental Science & Technology* 46 (10), S. 5497–5503. DOI: 10.1021/es2040009.

Kiefer, Nadine; Nichterlein, Moritz; Reiß, Fabienne; Runge, Madeleine; Biermann, Ulf; Wieland, Thomas et al. (2023): Eluates from façades at the beginning of their service time affect aquatic and sediment organisms. In: *The Science of the total environment* 906, S. 167531. DOI: 10.1016/j.scitotenv.2023.167531.

Schoknecht, Ute; Gruycheva, Jana; Mathies, Helena; Bergmann, Hannelore; Burkhardt, Michael (2009): Leaching of biocides used in façade coatings under laboratory test conditions. In: *Environmental Science & Technology* 43 (24), S. 9321–9328. DOI: 10.1021/es9019832.
